# COVID-19 vaccination uptake and safety profile among germline *BRCA1* and *BRCA2* pathogenic variant carriers in Singapore

**DOI:** 10.1186/s13053-023-00248-2

**Published:** 2023-04-12

**Authors:** Zewen Zhang, Nur Diana Binte Ishak, Frances Victoria Fajardo Que, Zi Yang Chua, Sock Hoai Chan, Jianbang Chiang, Joanne Ngeow Yuen Yie

**Affiliations:** 1 Cancer Genetics Service, Division of Medical Oncology, National Cancer Centre Singapore, 11 Hospital Crescent, Singapore, 169610 Singapore; 2 Lee Kong Chian School of Medicine, Nanyang Technological University, Singapore, 308232 Singapore

**Keywords:** Genetics, *BRCA1*, *BRCA2*, COVID-19, Vaccination

## Abstract

**Background:**

Although Singapore is one of the highest vaccinated countries in the world, vaccine hesitancy remains in a subpopulation, including individuals with cancer predisposition syndromes. At the Cancer Genetics Service National Cancer Centre Singapore, we see patients with germline genetic alterations, most being *BRCA1/2* pathogenic/likely pathogenic variant (PV/LPV) carriers. While reported safe for cancer patients, there are limited studies addressing the safety profile and outcomes of COVID-19 vaccination among individuals with germline PV/LPV in cancer predisposition genes such as *BRCA1/2*. This study aims to evaluate the outcomes of COVID-19 vaccination among germline PV/LPV carriers in *BRCA1/2*.

**Methods:**

We conducted a phone call survey of COVID-19 vaccination uptake and toxicity in a prospective cohort of 189 participants with germline *BRCA1/2* PV/LPV between 1st Sept 2021 and 30th Sept 2021. We collected demographics data including gender, race, age, history of cancer, types of cancer, and number of cancers. Statistical difference in baseline demographics between responders with history of cancer and those without were assessed using Chi-square, Fisher’s exact and independent t-test analysis. Logistic regression was used to evaluate effect of demographics on the occurrence of post-vaccination side effects.

**Results:**

Among 189 *BRCA1/2* PV/LPV carriers responded, 97 carried PV/LPV in *BRCA1* and 92 in *BRCA2*. Majority were vaccinated (89.5%) and had completed the two-dose vaccine schedule, with 7 (4.1%) received only one dose. The most common post-vaccination side effects was myalgia (56.5%) followed by fever (40.2%), headache (16.3%) and fatigue (11.2%). There were no major severe side events. Evaluation by logistic regression showed that the occurrence of side effects was not affected by PV/LPV gene (*BRCA1* or *BRCA2*), gender, race, age or history of cancer.

**Conclusion:**

The post-vaccination side effects profile among individuals with germline PV/LPV in *BRCA1/2* is consistent with the Singaporean general population, hence recommendations for COVID-19 vaccination for these individuals should not differ from non-carriers and should be encouraged by their healthcare providers.

## Background

Although the course of Coronavirus disease 2019 (COVID-19) is mild to moderate in most individuals without comorbidities, patients with cancer are more vulnerable to complications and mortality from COVID-19, demonstrating greater mortality rates at 1.5 to 2 times compared to non-cancer patients [1, 2]. One of the many factors postulated to be associated with increased susceptibility among cancer patients is the immune-compromised state attributed by malignancy-driven processes or treatment related complications [3–5]. As functional innate and adaptive immune systems are critical for control and clearance of severe acute respiratory syndrome coronavirus 2 (SARS-CoV-2) [6, 7], it is conceivable that acquired or constitutional immune-related deficiencies among cancer patients could contribute to their increased risk of COVID-19 severity and complications. This includes cancer patients with inherited DNA repair syndromes that are also associated with immunodeficiencies such as ataxia-telangiectasia, Nijmegen breakage syndrome and Fanconi anemia [8, 9] .

Approximately 5–10% of the cancer patient population harbour germline genetic alterations in cellular DNA repair genes associated with predisposition to malignancies [8, 9], of which *BRCA1/2* are often reported and primarily associated with breast and ovarian cancers. *BRCA1/2* are tumour suppressor genes involved in the Fanconi anemia pathway, a critical component of cellular DNA homologous recombination. A few studies have suggested that individuals with Fanconi anemia carry lower B cells, CD4^+^ T cells and natural killer cells compared to the normal range [10, 11]. As COVID-19 vaccines have been shown to be effective for immunocompromised and cancer patients [12–16], it is reasonable to conjecture that individuals with inherited DNA repair deficiencies would especially benefit from COVID-19 vaccination.

While COVID-19 vaccination has been demonstrated to be safe for cancer patients [17, 18], there are limited reports specifically addressing the safety profile and outcomes among individuals with germline pathogenic/likely pathogenic variants (PV/LPV) in cancer predisposition genes or inherited DNA repair disorders. At present, vaccine hesitancy remains a global issue and even among our highly vaccinated Singaporean population, there are individuals with cancer and cancer predisposition syndromes who harbour concerns on the safety profile and efficacy of the COVID-19 vaccines, as clinical trials for vaccines had excluded these patient populations. Currently, there are no data to address these concerns hence in this study, we aim to evaluate the safety profile of COVID-19 vaccines among 265 individuals with germline PV/LPV in *BRCA1/2*. We believe that our report will help address concerns on the safety of COVID-19 vaccination among individuals with cancer predisposition syndromes especially hereditary breast and ovarian cancer syndrome, which affects a remarkable proportion of the cancer population, so that they may consider the benefits of COVID-19 vaccination.

## Methods

This study was approved by the SingHealth Centralised Institutional Review Board (IRB: 2021–2593). Eligible participants were recruited between 1st September 2021 to 30th September 2021 from the Cancer Genetics Service (CGS) at National Cancer Centre Singapore (NCCS). A phone call survey on COVID-19 vaccination was conducted by three research coordinators and genetic counsellors on 265 individuals previously underwent genetic testing at CGS and received a germline *BRCA1/2* PV/LPV result. All participants were above 21 years of age and fulfilled the following criteria: a) Singapore resident, b) carrier of *BRCA1/2* germline PV/LPV identified through genetic testing performed by a Clinical Laboratory Improvement Amendments/ College of American Pathologists (CLIA/CAP) certified laboratory, and c) with or without a history of cancer. Individuals who declined the survey or did not respond after five calls were removed from the study.

All responders completed our Questionnaire (Appendix A) to evaluate their participation in the National COVID-19 Vaccination Programme in Singapore. Participants were required to report their choice of vaccine, dose, date of vaccination and to provide a “Yes” or “No” response to the occurrence and type of side effects post-vaccination. Symptoms in our questionnaire were based on the expected side effects from the Singapore’s Ministry of Health frequently asked questions on COVID-19 vaccine [19]. Reports of additional side effects not included in the Questionnaire list were manually recorded. The side effects were self-reported by participants and were not categorized by grades. Unvaccinated participants were invited to disclose their reason for declining vaccination. Participants were also asked to declare their history of COVID-19 infection. Demographics data including gender, race, age, history of cancer, types of cancer, and number of cancers were retrieved from their medical records (Table 1).

**Table 1:** Demographics of Respondents and Vaccination details

Completed COVID questionnaire (***n*** = 189)			***p***-value
	Personal history of cancer (*N* = 130)	Nil history of cancer (*N* = 59)	
**Gender**			
Female	124 (95%)	38 (64%)	< 0.001^+^
Male	6 (5%)	21 (36%)	
**Race**			
Chinese	84 (65%)	44 (75%)	0.152^*^
Malay	21 (16%)	7 (12%)	
Indian	13 (10%)	3 (5%)	
Others	12 (9%)	4 (7%)	
Unspecified	0	1 (2%)	
**Age, years**			
Median (Range)	53.5 (28–79)	42.5 (22–77)	< 0.001^#^
Mean (SD)	53.5 (±11.7)	45.7 (±14.0)	
**Germline pathogenic gene**			
*BRCA1*	65 (50%)	32 (54%)	0.531^+^
*BRCA2*	65 (50%)	27 (46%)	
**Types of cancer**			
Breast	78 (60%)	NA	
Ovarian	28 (22%)		
Others	7 (5%)		
More than 1 cancer	16 (12%)		
Unspecified/No cancer	1 (1%)		
**COVID Vaccination**			
**Yes**	116 (89%)	54 (92%)	0.796^+^
1 dose	11 (8%)	1 (2%)	
2 doses	105 (81%)	51 (86%)	
Unspecified	0 (0%)	2 (3%)	
**No**	14 (11%)	5 (8%)	
**Previous COVID-19**			
Yes	2 (2%)	0 (0%)	1.000^*^
No	125 (96%)	59 (100%)	
Unspecified	3 (2%)	0 (0%)	
**Vaccinated individuals**			
**Type of Vaccination**	***N*** **= 116**	***N*** **= 54**	0.806^*^
Pfizer	97 (84%)	47 (87%)	
Moderna	13 (11%)	7 (13%)	
Sinovac	3 (3%)	0 (0%)	
Unspecified	3 (3%)	0 (0%)	
**Side effects post vaccination**			
**Yes**	64 (55%)	28 (54%)	0.740^+^
1 side effect	36 (31%)	15 (28%)	
2 side effects	20 (17%)	14 (26%)	
Multiple (> 2) side effects	8 (7%)	0 (%)	
**No**	50 (43%)	25 (46%)	
Unreported	2 (2%)	0 (%)	

*Based on Fisher’s exact test

^+^Based on Pearson Chi-Square test

^#^Based on t-test

Our primary outcome was the proportion of individuals with germline *BRCA1/2* pathogenic variants who received the COVID-19 vaccine, and our secondary outcome was to evaluate the proportion and type of side effects after vaccination. Statistical difference in baseline demographics between participant with history of cancer and those without were assessed using Chi-square, Fisher’s exact and independent t-test analysis using SPSS (28.0.1.1). The effect of participant demographics on the occurrence of post-vaccination side effects were evaluated using logistic regression.

## Results

Of the 265 pathogenic *BRCA1/2* PV/LPV carriers surveyed, 189 (71.3%) participated in the study (Table 1), of whom 97 carried PV/LPV in *BRCA1* and 92 in *BRCA2*. Majority of responders were vaccinated (89.5%, 170/189), of whom 144 (84.7%) received Pfizer-BioNTech/Comirnaty, 20 (11.8%) received Moderna/Spikevax, three (1.8%) received Sinovac-CoronaVac, and three (1.8%) were unable to recall type of vaccines received (Table 1). Majority (92.3%) of vaccinated responders had completed the two-dose vaccine schedule, with 7 (4.1%) having received only one dose. Of the seven, five planned to receive the second dose as scheduled, one declined the second dose for reasons undisclosed, and one had history of COVID-19 infection and was advised that the second dose was not required at the time of questionnaire.

Among the 19 unvaccinated responders, the most common reason for declining vaccination was active cancer treatments (31.6%, 6/19). Three (15.8%) responders felt uncertain as they had not consulted a physician, whereas three (15.8%) were advised to wait for other types of vaccines in view of allergies to messenger ribonucleic acid (mRNA) vaccines (Table 2). One unvaccinated participant had a history of COVID-19 infection.

**Table 2:** Reasons for not receiving COVID-19 vaccination

Unvaccinated	*N* = 19
Active treatment including chemotherapy	6
Have not consulted Physician	3
Just received Physician’s recommendation	1
Vaccine not recommended	1
Allergic/advised to wait	3
Have not had time to do it	1
Does not believe in the vaccine	1
Prefer not to disclose	2
Unknown	1

Among all vaccinated responders, 55.1% (92/167) developed post-vaccination side effects, the most common being myalgia (56.5%). Other side effects include fever (40.2%), headache (16.3%), fatigue (11.2%), and rashes (6.5%) (Table 3). Overall, 51 (55.4%) responders experienced one side effect, 33 (35.9%) with two side effects, and the remaining (8.7%) experiencing more than two side effects post vaccination. There were no major severe side events of Common Terminology Criteria for Adverse Events (CTCAE) grade 3 and above. Univariate analysis was performed to examine associations of demographical variables with personal cancer history and post vaccination side-effects without. A chi-square test of independence revealed a significant difference between gender and personal cancer history, X^2^(1, 188) = 32.545, *p* < 0.001. Women were more likely than men to have a personal history of cancer. Age of participants with personal history of cancer (Mean = 53.5, SD = 11.71) were significantly higher t (186) = 3.982, *p* < 0.001 than age of participants without cancer (Mean = 45.7, SD = 13.96) (Table 1). No significant associations were found between post-vaccination side effects and demographical variables (Table 4).

**Table 3:** Side effects experienced post vaccination

Individuals with side effects post vaccination	***N*** = 92
Headache	15 (16.3%)
Fever	37 (40.2%)
Body aches (Myalgia)	52 (56.5%)
Rashes	6 (6.5%)
**Others:**	33
Chills	6
Itch	1
Fatigue	11
Flushing	1
Swollen arm, hands	6
Nausea	3
Swollen lymph node (breast, neck)	3
Eczema flare, allergy	2
Breathing difficulty	1
Affected period cycle	1
Patients without side effects	**5**

**Table 4:** Association of demographic factors with the occurrence of side effects post vaccination

	Side Effects Post Vaccination		***p***-value
	No (*N* = 75)	Yes (*N* = 92)	
**Gender**			
Female	62 (83%)	81 (88%)	0.378^+^
Male	13 (17%)	11 (12%)	
**Race**			
Chinese	56 (75%)	60 (65%)	0.349^+^
Malay	8 (11%)	14 (15%)	
Indian	7 (9%)	7 (8%)	
Others	4 (5%)	11 (12%)	
Unspecified	0	1 (1%)	
**Age, years**			
Mean (SD)	52.9 (±12.1)	49.9 (±12.9)	0.134
**Germline pathogenic gene**			
*BRCA1*	38 (51%)	48 (52%)	0.877^#^
*BRCA2*	37 (49%)	44 (48%)	
**History of Cancer**			
No	25 (33%)	28 (30%)	0.740^+^
Yes	50 (67%)	64 (70%)	

^+^Based on Pearson Chi-Square test

^#^Based on independent t-test

## Discussion

Although not mandatory, Singapore offers free COVID-19 vaccination under the National Vaccination Programme for citizens and long-term residents during the pandemic. Globally, Singapore is third country after Qatar and Brunei with the highest proportion of population vaccinated (90.9%) [19, 20]. Nevertheless, a small fraction of our population, including cancer patients, remained hesitant on COVID-19 vaccination primarily due to vaccine safety and efficacy concerns. Under the Singapore healthcare framework, individuals with cancer on active treatment or those in remission are given recommended for COVID-19 vaccine if deemed eligible by their primary physician. Patients on active treatment are considered to be at least moderately immunocompromised and are further encouraged to receive a third dose of vaccine 2 months after their second dose [21]. Conceivably, individuals with inherited genetic disorders associated with impaired immune function will also stand to benefit from these vaccination strategies.

In our evaluated cohort of *BRCA1/2* germline PV/LPV carriers, a greater proportion of cancer patients were female (95%) compared to non-cancer patients (64%), and cancer patients were older than non-cancer patients. This is in agreement with literature, where male with *BRCA1/2* PV/LPV are often silent carriers [22]. Consistent with the Singapore general population (data up to December 2021) [19, 21], our cohort of 189 germline PV/LPV carriers in *BRCA1/2* revealed a 90% COVID-19 vaccination uptake rate, predominantly for Pfizer-BioNTech/Comirnaty or Moderna/Spikevax mRNA vaccines and had completed the two-dose primary vaccination schedule at the time of our survey.

At least half of the responders developed post-vaccination side effects, most frequently myalgia followed by fever, which is consistent with side effects typically observed following flu vaccinations. This profile is similar to that reported in other COVID-19 vaccination studies on cancer patient cohorts [15, 16]. None of the demographic variables tested predicted for the occurrence of side effects. There were no major adverse events, such as anaphylaxis or hospitalizations, reported by our study responders. The nature of side effects post-vaccination from our survey were similar to the vaccine studies reported on the Centre for Disease Control and Prevention (CDC) website, with the most common being fatigue, headache and myalgia regardless of age [23]. Although participants were not asked grade of the side effects, the reporting of events in a categorical manner allowed us to compare the observations reported by CDC. The frequency of side effects on CDC was in the range of 70.6 to 82.8% depending on age groups for the Pfizer-BioNTech COVID-19 vaccine and was approximately 55% in our cohort with a median age of 50 years old. In the age group between 18 and 55 years of age the percentage of individuals reported to have local reactions such as redness and swelling was between 4.5 to 6.3%, comparable with our participants with rashes at 6.5%. Reported “fever and chills” in the CDC groups was between 3.7 to 35.1%, while 40.2% of our participants experienced fever. Higher number of headache was reported at 41.9% in the CDC group while 16.3% of our participants had headache post vaccination. Our participants reported lower percentage of fatigue at 11.2% while CDC was between 47.4 to 59.4%. Our results were also similar to a local study evaluating the side effects after COVID-19 vaccination among 1704 healthcare workers (HCW) in Singapore. The HCW responded to an online survey after receiving two doses of Pfizer BioNTech/Comirnaty, most documented side effects of fever (9.3–44.7%), headache (18.8–41.7%), myalgia (30.1–51.9%) and none reported severe reactions, such as anaphylaxis [24].

A clinically relevant side effect of COVID-19 vaccination is the enlargement of axillary lymph nodes on the ipsilateral arm of vaccination site. In a literature review of 15 studies, the reported incidence of regional lymphadenopathy was 14.5–53.0% and persisted for more than 6 weeks in 29.0% of recipients [25]. Most lymphadenopathies (32%) in the study were detected on imaging whereas only 1.8% were self-reported. Our cohort had 3 (1.8%) vaccinated responders with reported lymphadenopathy. As part of regular surveillance for breast cancer, PV/LPV carriers of *BRCA1/2* are recommended for annual breast magnetic resonance imaging and breast mammogram as early as age 25 years and hence, it may be prudent for physicians to advise COVID-19 vaccine recipients to defer imaging scans minimally a few weeks post-vaccination to avoid false positives for pathological conditions.

Our study is limited by the small sample size, mostly due to a notable (28.3%) non-responding rate to our phone call survey, and lack of a control cohort without germline PV/LPV in *BRCA1/2* for statistical evaluation. As side effects were self-reported by responders, our assessment could be an underestimate due to subjectivity across individuals and the questionnaire used precluded evaluation of any temporal relationship between side effects and vaccine dose. Nevertheless, our observations on the side effects profile were consistent with the general Singapore population and other cancer patient cohorts, defined by predominantly mild and self-limiting effects such as myalgia, fever and headache with no severe side effects.

## Conclusions

This study evaluated the safety profile of COVID-19 vaccination on a cohort of individuals with germline PV/LPV in *BRCA1/2* and showed that the post-vaccination side effects profile experienced were similar with the general population with no major side effects. As such recommendations with regards to COVID-19 vaccination for eligible individuals with germline PV/LPV in *BRCA1/2* should not differ from non-carriers and should be encouraged by their healthcare providers.

## Data Availability

The datasets used and/or analysed during the current study are available from the corresponding author on reasonable request.

## APPENDIX

### Appendix A: COVID Vaccination Questionnaire

Name:

IC:

Date of clinic visit:

1. Have you had your COVID Vaccination **(Yes/No)** – if no please proceed to Question 4
2. When did you receive your vaccination?

#1 ____________ dd/mm/year.

#2____________ dd/mm/year

3) Name of COVID Vaccine **(Pfizer/Moderna/Sinovac/Others/Unsure)**
4) Reason for not receiving vaccination: _______________
5) Have you experienced any side effects from the vaccination? (Yes/No)

If “Yes” please select the appropriate answers, you may choose more than one

A) Headache
B) Fever
C) Body aches
D) Rashes
E) Others__________ (please specify)

6) Have you ever been tested positive for COVID? (Yes/No)

If “Yes”, when was it ___________ (dd/mm/year).

## Abbreviations

COVID-19: Coronavirus disease 2019
PV: Pathogenic variant
LPV: Likely pathogenic variant
CGS: Cancer Genetics Service
NCCS: National Cancer Centre Singapore
HBOC: Hereditary Breast and Ovarian Cancer syndrome
CLIA: Clinical Laboratory Improvement Amendments
CAP: College of American Pathologists
mRNA: Messenger ribonucleic acid
CTCAE: Common Terminology Criteria for Adverse Events
CDC: Centre for Disease Control and Prevention
